# Temperature-Dependent Microstructure and Tribological Performance of Boride Layers Formed on 40 Kh Steel Using Boric Acid-Based Boriding

**DOI:** 10.3390/ma18184342

**Published:** 2025-09-17

**Authors:** Laila Sulyubayeva, Daryn Baizhan, Nurbol Berdimuratov, Dastan Buitkenov, Balym Alibekova

**Affiliations:** 1 Research Center Surface Engineering and Tribology, Sarsen Amanzholov East Kazakhstan University, Ust-Kamenogorsk 070000, Kazakhstan; lsulyubayeva@gmail.com (L.S.); daryn.baizhan1@gmail.com (D.B.); dbuitkenov@vku.edu.kz (D.B.); balymalibekova304@gmail.com (B.A.); 2 Research School of Physical and Chemical Sciences, Shakarim University, Semey 071412, Kazakhstan

**Keywords:** boriding, boric acid, microstructure, wear, microhardness

## Abstract

Boriding is widely used in various industries due to the unique combination of high mechanical, corrosion, and tribological properties of boride layers formed on the surface of steel components. In this work, the powder boriding of 40 Kh steel was investigated in a closed capsule using a specially prepared powder mixture containing boric acid as the boron source. Boriding was carried out in a furnace at 850, 900, and 950 °C for 10 h. The resulting boride layers were characterized using scanning electron microscopy (SEM) and X-ray diffraction (XRD), which confirmed that all three coatings consist exclusively of the Fe_2_B phase. It was found that with increasing temperature, the thickness of the boride layer increased from 68 μm to 160 μm. The tribological properties were evaluated using the pin-on-disk method, followed by analysis of the wear surfaces using optical profilometry and SEM. The most significant reduction in wear rate was observed at 850 °C, where the wear decreased by a factor of 4.2—from 8.471 × 10^−5^ to 1.999 × 10^−5^ mm^3^·N^−1^·m^−1^. In addition, the hardness increased fivefold compared to the untreated material. These results demonstrate the high potential of diffusion boriding for enhancing the operational performance of parts subjected to severe wear conditions.

## 1. Introduction

One of the key factors determining the operational performance and durability of engineering parts and tools is the set of physical and mechanical properties of their surface layer. It is in the near-surface zone that the highest mechanical stresses are concentrated and where fatigue, wear, and corrosion processes are initiated and developed [1]. It is well known that the degradation of working surfaces due to wear significantly reduces the technical performance of products and may ultimately lead to complete failure. An effective approach to improving the service life of components is to modify their surface layer by forming coatings with tailored properties, such as increased hardness, wear resistance, and corrosion resistance. The creation of such functional layers makes it possible to considerably enhance the reliability and lifetime of parts without the need to replace the bulk material [2,3].

Among the different approaches, chemical–thermal treatment (CTT) is one of the most studied and widely applied technologies for modifying the surface layers of engineering components [4]. Within this group, boriding is of particular interest because of the exceptionally high hardness of the resulting boride layers [5]. Boriding is a diffusion-based process in which the surface layer of a part or tool is saturated with boron atoms from a boron-containing medium at temperatures between 800 and 1000 °C [6]. Despite the limited solubility of boron in α-Fe, once a critical concentration is reached at the surface, boride phases—primarily FeB and Fe_2_B—are formed [7]. The subsequent increase in the thickness of the hardened layer is achieved by inward diffusion of boron into the substrate. Both the thickness and the phase composition and morphology of the boride layer are determined by a combination of factors, including treatment temperature and duration, the boron concentration in the saturating medium, and the chemical composition of the substrate [8,9,10].

Various technological approaches to boriding are used in industry, differing in the saturation mechanism, the nature of the medium used, and the temperature–time regime. One of the most common methods is gas boriding [11], carried out in furnaces at temperatures of 800–850 °C in an atmosphere of boron-containing gases, such as diborane (B_2_H_6_), boron trichloride (BCl_3_) or trimethylborane, diluted with inert or reducing gases (hydrogen, argon, ammonia or nitrogen) [12]. During the boriding process, these compounds dissociate to form atomic boron, which diffuses into the metal substrate at a temperature of about 850 °C (pressure of about 25 kPa). This results in the formation of a boride layer up to 0.2 mm thick with a high hardness of up to 2000 HV [13]. Despite the effectiveness of the method, gas boriding has limited application due to the high toxicity and explosiveness of the reagents used in the process [14].

Liquid boriding is typically carried out in molten chloride-based baths containing boron-bearing components such as boron trichloride (BCl_3_), ferroboron, and boron carbide (B_4_C), with the addition of chemical reducing agents (e.g., Al, Si, Ti) [15]. Depending on the melt composition, either single-phase (Fe_2_B) or two-phase (FeB + Fe_2_B) boride layers can be obtained [16]. In single-phase coatings composed of Fe_2_B, the distribution of residual stresses is more favorable, while in two-phase coatings (FeB + Fe_2_B), a sharp stress gradient is observed at the interphase boundary. The stresses generated within the FeB phase are predominantly tensile, whereas those in the Fe_2_B phase are compressive [17]. The presence of tensile stresses in FeB critically reduces the coating’s plasticity. As a result, even under minor bending, compressive, or especially impact loads, the surface layer is prone to cracking and delamination. Therefore, the formation of single-phase Fe_2_B coatings is considered most advantageous for ensuring the durability and mechanical reliability of the boride components [18].

If the steel contains alloying elements such as chromium or molybdenum, borides of these elements (e.g., CrB, Mo_2_B) can form within the boride layer, exhibiting hardness values of up to 25–30 GPa [19]. The presence of such superhard phases significantly enhances the wear resistance of the material and enables boronized parts to perform effectively under conditions of high contact loads and severe friction [20]. For example, Martínez-Nopala et al. [21] demonstrated a substantial reduction in erosion wear of boronized AISI D2 steels exposed to solid particle impact, further confirming the potential of boriding technology for extending the service life of engineering components.

In addition to volumetric methods, local boriding is widely applied using boron-containing pastes that are coated onto selected areas of the product surface. These pastes typically consist of boron carbide (B_4_C) mixed with borax (Na_2_B_4_O_7_) and sodium fluoride (NaF), while a mixture of acetone and adhesive serves as a binder. Following application, the coated part is sealed in a container and subjected to heat treatment at approximately 950 °C for 3–4 h [22]. As reported in [14], the maximum calculated diffusion coefficient of boron is achieved at 950 °C with a paste thickness of 5 mm. Under these conditions, the surface layer of steels such as AISI 1045 can be hardened to a thickness of ~100 µm, thereby markedly improving wear resistance in zones exposed to intensive friction.

Powder boriding has also gained significant industrial importance. In this process, parts undergo thermochemical treatment in boron-containing powders—such as amorphous boron, boron carbide, ferroboron, and their mixtures—at temperatures of 850–950 °C. Makuch et al. [23] demonstrated that two-phase boride coatings (FeB + Fe_2_B) produced via powder-pack boriding exhibit parabolic growth kinetics, high microhardness values (up to 1800 HV_0.05_), and enhanced surface durability. This approach is particularly effective for reinforcing large-scale or geometrically complex components, since it enables local and selective saturation of the surface with boron. Traditional activators of the diffusion process include fluorine-containing additives such as NaF, KF, NH_4_F, as well as tetrafluoroborates (NaBF_4_ and KBF_4_), which promote the formation of volatile boron species and accelerate boron transfer to the substrate [24,25,26,27]. However, these compounds are highly toxic and require stringent safety measures. In contrast, less toxic alternatives such as boric acid (H_3_BO_3_) and borax (Na_2_B_4_O_7_) have emerged as promising activators, offering effective boron transport with lower environmental risk.

The aim of this work is to establish an eco-friendly powder boriding route for 40 Kh steel using boric acid–based activators, with a focus on obtaining single-phase Fe_2_B layers and evaluating their microstructural, mechanical, and tribological performance at 850–950 °C.

## 2. Materials and Methods

The widely used medium-carbon structural 40 Kh steel was selected as the substrate material for powder boriding. Its chemical composition is presented in Table 1.

Prior to boriding, the steel samples (20 × 20 × 10 mm^3^) were mechanically ground and ultrasonically cleaned in ethanol to remove surface contaminants. A specially prepared boriding powder mixture was used as the saturating medium. The composition and particle sizes of the powders are presented in Table 2.

The powders were mechanically mixed for 30 min in a planetary ball mill (Retsch PM100, Haan, Germany) to ensure homogeneity. The prepared samples were placed in a cylindrical capsule made of heat-resistant AISI 304 steel (Ø30 mm × 50 mm), fully filled with the powder mixture, hermetically sealed by welding, and subjected to heat treatment in a high-temperature furnace SNOL 30/1300 (SNOL, Utena, Lithuania). The heating rate was 10 °C/min, and the process was carried out at 850, 900, and 950 °C for 10 h. After treatment, the capsule was removed from the furnace and cooled in air at room temperature.

The phase composition of the boride layers was examined by X-ray diffraction (XRD) using a Shimadzu XRD-6000 diffractometer (Kyoto, Japan) equipped with a monochromatic copper anode radiation source (CuKα, λ = 1.54056 Å). The measurement parameters were as follows: acceleration voltage 45 kV, beam current 30 mA, scanning step 0.02°, scanning range 2θ = 20–90°, and a signal accumulation time of 0.5 s per step. Phase identification was carried out using the PDF-4+ database (ICDD, Newtown Square, PA, USA), and quantitative full-profile analysis was performed with POWDER CELL 2.4 software. The characteristics of the borated samples were studied using microstructural analysis, elemental composition, microhardness measurements and tribological tests. Microstructural studies were performed on transverse sections of the borated samples prepared using a standard metallographic technique, including grinding, polishing and etching with a nitric acid-based reagent. Microstructural analysis was performed using SEM3200 (Guangzhou, China) scanning electron microscopy.

The methods for measuring the microhardness of the boride layers met the requirements of DIN EN ISO 14577-1 [28] and ASTM E2546 [29]. To determine the hardness distribution across the thickness of the boride layer, a FISCHERSCOPE^®^ HM2000S microhardness tester (Helmut Fischer GmbH, Sindelfingen, Germany) equipped with Vickers indenters was used. The measurements were taken under a load of 0.98 N applied for 10 s. The measurements were performed on a transverse section of the samples—from the surface of the boride layer to the metal matrix. The hardness profile was constructed based on the results obtained at points uniformly distributed across the thickness. To increase the reliability and reproducibility of the results, three independent measurements were taken at each point in different areas, after which the average microhardness value was calculated.

**Figure 1:**
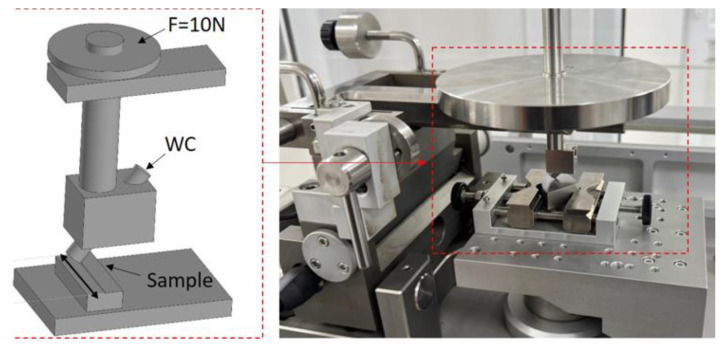
Tribological testing of the boride layer during reciprocating motion.

Tribological tests of the borated specimens and the original specimen were investigated under conditions of dry sliding of a WC pin (6 mm diameter) on the specimen surface using the pin-on-disk test method (tribometer Anton Paar TRB^3^, Graz, Austria) with reciprocating motion of the tribometer [30] (ASTM G133). Wear tests were carried out at room temperature under a normal load of 10 N with a sliding frequency of 1 Hz along a linear trajectory with a full amplitude of 14 mm over a sliding distance of 100 m. After the wear tests, wear traces, thickness and area of the track part were investigated using a surface profilometer (Taylor Hobson Ltd., Leicester, UK; ISO 4287 standard [31]) Figure 1.

The volumetric wear coefficient of worn samples was calculated by multiplying the length and cross-sectional area of the wear trace (1):

(1) WRs=S×lL×Fn

where:

▪ *S* is the worn track section mm^2^;
▪ *l* is the full amplitude mm;
▪ *L* is the total measurement distance m;
▪ *F_n_* is the normal load N;
▪ *WR_s_* is the wear rate of the moving sample mm^3^·N^−1^·m^−1^.

The wear intensity values were calculated by dividing the volume by the applied load and the sliding distance. The worn surfaces were also examined using SEM. Elemental analysis of the sample surfaces before and after the wear test was performed using EDS analysis.

## 3. Results and Discussion

**Figure 2:**
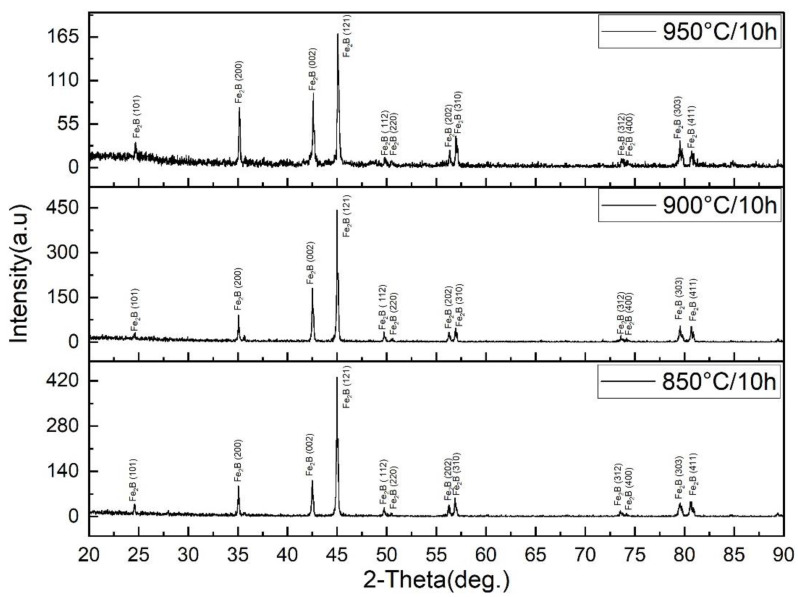
XRD patterns of borided 40 Kh steel obtained at 850, 900, and 950 °C for 10 h.

The phase composition of the boride layers was examined using X-ray diffraction (XRD), and the obtained patterns are presented in Figure 2. For all investigated boriding conditions (850, 900, and 950 °C, 10 h), the diffraction peaks correspond exclusively to the Fe_2_B phase, with the most intense reflections detected at (002), (121), (200), and (112) planes, in agreement with the standard JCPDS card No. 35-1333 [32,33]. No additional peaks related to FeB or other boride phases were observed, confirming that the boriding process in the boric acid/borax-based powder mixture consistently produced a single-phase Fe_2_B layer across the entire temperature range. The relative intensity of Fe_2_B reflections increases with temperature, particularly for the (002) and (121) peaks, indicating improved crystallinity and preferential orientation of Fe_2_B grains at higher boriding temperatures. This trend is consistent with the SEM observations of denser and more compact Fe_2_B microstructures at 900–950 °C. The absence of FeB, which is typically associated with brittleness and microcracking, is a key advantage of the present process, as it ensures the formation of Fe_2_B layers combining high hardness with superior toughness.

**Figure 3:**
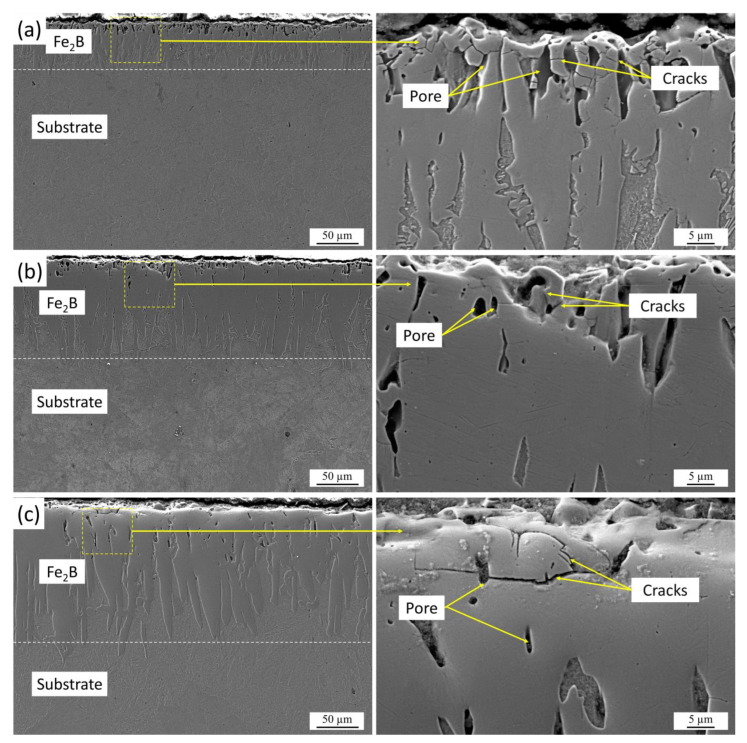
SEM images of cross-sections of borided 40 Kh steel at: (**a**) 850 °C, (**b**) 900 °C, and (**c**) 950 °C for 10 h.

The morphology of the boride layers and their average thickness were investigated using scanning electron microscopy (SEM). Cross-sectional micrographs of 40 Kh steel after boriding at 850, 900, and 950 °C for 10 h (Figure 3) clearly demonstrate the formation of dense, compact layers with a characteristic needle-like morphology. The elongated Fe_2_B crystals grow perpendicularly from the surface and form a continuous structure with strong adhesion to the substrate. Such morphology is typical of diffusion-controlled boriding and indicates effective penetration of boron into the steel matrix. The diffusion of boron results in the development of a boride zone, followed by a transitional sublayer that can be interpreted as a solid solution of boron in the ferritic matrix, providing a gradual compositional transition between the coating and the base metal. XRD analysis confirmed that, under all treatment conditions, the boride layers consist exclusively of the Fe_2_B phase. The absence of FeB is noteworthy, as this phase, despite its very high hardness, is inherently brittle and often associated with crack initiation and premature spallation of the coating under mechanical stress. By contrast, Fe_2_B exhibits a superior balance of hardness and toughness, enabling it to resist both abrasive and fatigue wear mechanisms. This phase stability is, therefore, critical to ensuring the structural integrity and durability of the boride layers. The SEM images support this conclusion, showing uniform Fe_2_B morphology across all tested temperatures without evidence of phase stratification [34].

This growth behavior is consistent with the parabolic kinetics typically observed in diffusion-controlled processes, where layer thickness increases proportionally to the square root of time, while temperature exerts an exponential effect on diffusion coefficients. Importantly, the thickest layers obtained at 950 °C also contained a higher density of pores and microcracks. These defects can be attributed to volumetric mismatches and internal stresses associated with rapid boron diffusion and accelerated growth of needle-like Fe_2_B crystals.

**Figure 4:**
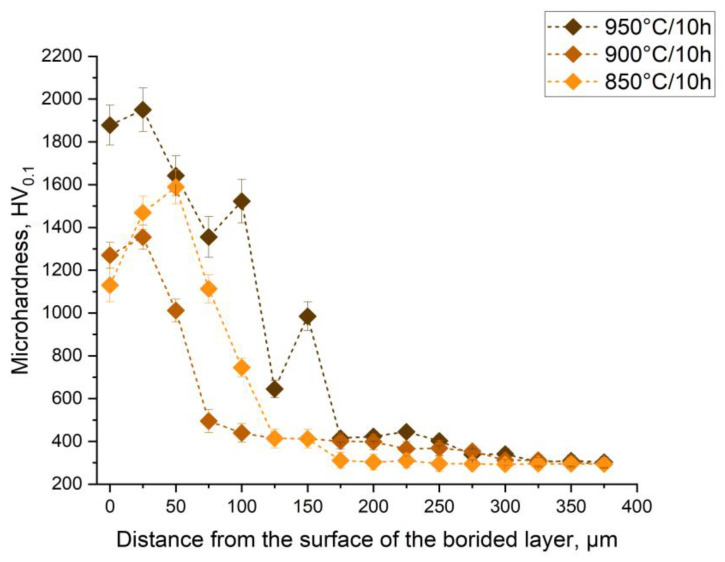
Microhardness of the cross-section of boride layers formed on 40 Kh steel during 10 h of heat treatment at: 850 °C, 900 °C, and 950 °C.

The microhardness values shown in Figure 4 reflect the characteristic variation of hardness across the cross-sections of boride layers formed on 40 Kh steel at 850, 900, and 950 °C for 10 h. These values represent averages obtained from a series of independent measurements in different areas of the cross-sections. The maximum microhardness near the surface reached ~1450 HV_0.1_ at 850 °C, ~1700 HV_0.1_ at 900 °C, and more than 2000 HV_0.1_ at 950 °C, which corresponds to the presence of the hard Fe_2_B phase confirmed by XRD [35]. All measured values significantly exceed the hardness of the base 40 Kh steel (~320 HV_0.1_), clearly demonstrating the effectiveness of diffusion boriding for improving surface properties.

**Figure 5:**
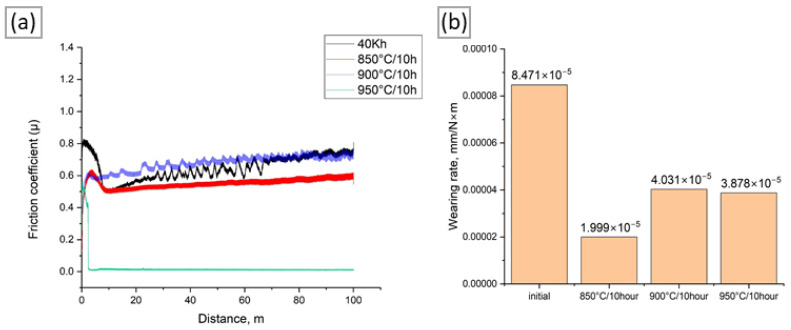
Tribological test results: (**a**) friction coefficient, (**b**) reduced wear.

As shown in Figure 5a, the untreated 40 Kh steel exhibited an initially high coefficient of friction (COF) exceeding 1.2, followed by stabilization at ~0.8 throughout the sliding path. This behavior is typical of abrasive wear accompanied by plastic deformation and micro-ploughing, which is explained by the ductile response of the steel surface. In contrast, the borided samples demonstrated a pronounced improvement in tribological performance. The specimen treated at 850 °C for 10 h showed a lower and more stable COF of ~0.6, while at 900 °C the COF further decreased to ~0.5. The lowest value was observed for the sample borided at 950 °C, where the COF stabilized at ~0.2, indicating the formation of a dense and hard Fe_2_B layer that effectively reduced tangential stresses in the contact zone.

These observations are consistent with the results of the specific wear rate measurements (Figure 5b). The untreated steel exhibited the highest wear rate of 8.471 × 10^−5^ mm^3^·N^−1^·m^−1^. After boriding, this value decreased more than fourfold, reaching 1.999 × 10^−5^ mm^3^·N^−1^·m^−1^ at 850 °C. At higher treatment temperatures, the wear rate increased slightly (to 4.031 × 10^−5^ and 3.878 × 10^−5^ mm^3^·N^−1^·m^−1^ at 900 and 950 °C, respectively). This effect can be attributed to the higher internal stresses and microcrack density in the thicker Fe_2_B layers formed at elevated temperatures. The reduction in both friction and wear after boriding is associated with the substantial increase in surface microhardness and the possible formation of secondary oxide films during sliding, which can act as a solid lubricant and further stabilize the tribological response.

**Figure 6:**
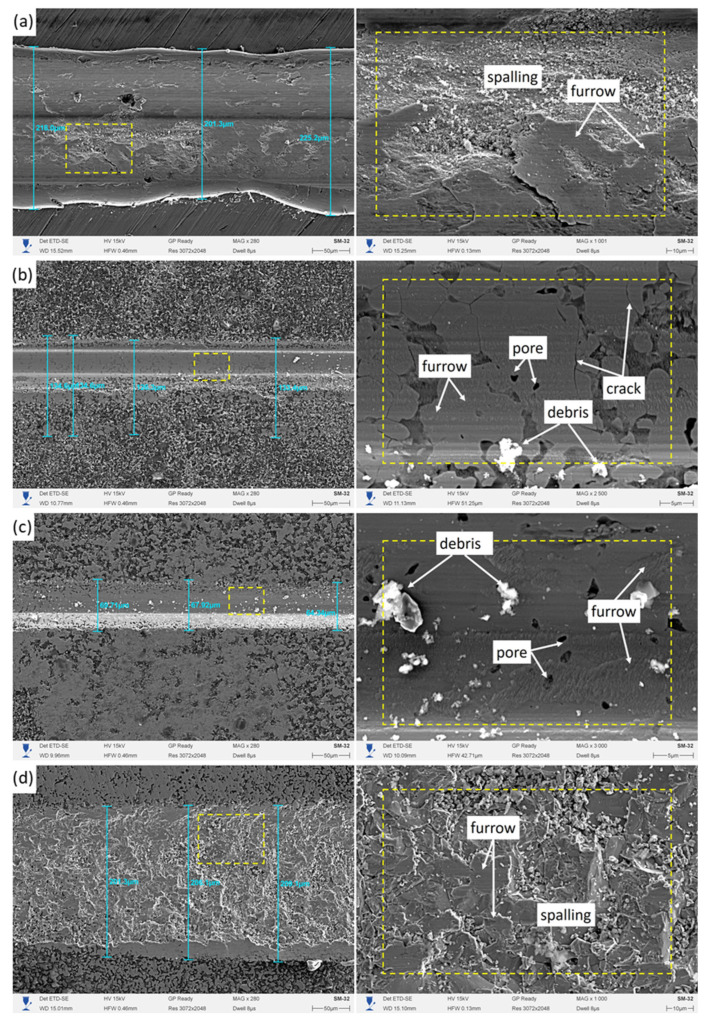
Wear marks on 40 Kh steel (**a**) and boride layers at: (**b**) 850 °C, (**c**) 900 °C and (**d**) 950 °C.

Microstructural analysis of worn surfaces (Figure 6) revealed significant differences in the dominant wear mechanisms depending on the boriding temperature. The untreated sample (Figure 6a) exhibited deep wear grooves, flaking, and microcracks oriented along the sliding direction, which are indicative of abrasive wear combined with fatigue damage. Such morphology is typical of surfaces subjected to cyclic contact stresses in the absence of a hardened protective layer.

After boriding at 850 °C (Figure 6b), the worn surface showed a clear reduction in the depth and density of wear grooves, although isolated microcracks and pores were still observed. This morphology suggests partial improvement in resistance to abrasive and fatigue wear due to the formation of a Fe_2_B-rich boride layer. The sample borided at 900 °C (Figure 6c) demonstrated further enhancement, with grooves becoming noticeably shallower and microcracks largely suppressed, resulting in a smoother and more uniform surface. This indicates the formation of a denser and more compact Fe_2_B layer capable of effectively redistributing contact stresses and delaying crack initiation.

By contrast, the sample borided at 950 °C (Figure 6d) displayed signs of a more complex wear mechanism, combining abrasive, adhesive, and fatigue components. Although XRD confirmed that only Fe_2_B was present, the increased layer thickness at this temperature led to higher internal stresses and microcrack density, which promoted localized delamination and flaking. The associated increase in microroughness facilitated interlocking with counterbody asperities, thereby intensifying stress concentration and initiating brittle fracture, even though the measured coefficient of friction (COF) was lower [36].

Thus, boriding significantly improves the tribological performance of 40 Kh steel by producing hardened Fe_2_B layers. However, the effectiveness of the treatment is governed not only by the thickness and hardness of the layer, but also by its microstructural homogeneity and defect density, which determine resistance to brittle fracture under cyclic contact loading.

## 4. Conclusions

This study demonstrated that diffusion boriding of 40 Kh steel at 850–950 °C for 10 h in a powder mixture activated with boric acid results in the formation of dense, single-phase Fe_2_B layers. XRD analysis confirmed the absence of FeB and verified that the boride layers consisted exclusively of Fe_2_B, characterized by elongated needle-like crystals strongly bonded to the substrate. The thickness of the boride layers increased systematically with temperature, from ~68 μm at 850 °C to ~160 μm at 950 °C, in agreement with parabolic diffusion-controlled growth kinetics.

The Fe_2_B layers exhibited exceptionally high hardness, reaching more than 2000 HV_0.1_ at 950 °C, which represents a nearly fivefold increase compared to untreated steel (~320 HV_0.1_). Tribological tests further revealed that boriding substantially reduced the coefficient of friction and wear rate. The most pronounced improvement was observed at 850 °C, where the wear rate decreased more than fourfold, while at 900 °C the Fe_2_B layer provided the most favorable balance between hardness, compactness, and defect density. At 950 °C, despite achieving the lowest COF (~0.2), increased porosity and microcrack density partially limited the wear resistance.

Overall, the results highlight that the performance of borided 40 Kh steel is governed not only by layer thickness and hardness, but also by phase purity, microstructural homogeneity, and resistance to brittle fracture. The exclusive formation of Fe_2_B using eco-friendly activators demonstrates a sustainable and effective approach for producing high-performance boride layers. This makes the proposed method highly promising for the surface strengthening of engineering components operating under severe wear conditions.

## Figures and Tables

**Table 1:** Chemical composition of 40 Kh steel (wt.%).

C	Si	Mn	Ni	S	P	Cr	Cu	Fe
0.36–0.44	0.17–0.37	0.5–0.8	up to 0.3	up to 0.035	up to 0.035	0.8–1.1	up to 0.3	about 97%

**Table 2:** Composition and particle size of the boriding powder mixture.

Components of Boriding Powder Mixture	Mixing Ratio (wt%)	Powder Particle Size (μm)
Boric acid (H_3_BO_3_)	40%	33.2
Borax (Na_2_B_4_O_7_)	10%	19.8
Silicon carbide (SiC)	50%	25.5

## Data Availability

The original contributions presented in this study are included in the article. Further inquiries can be directed to the corresponding author.
